# How to estimate health-related costs: economic aspect of healthy life style and its importance for PPPM

**DOI:** 10.1186/1878-5085-5-S1-A103

**Published:** 2014-02-11

**Authors:** Marko Kapalla, Juraj Kubáň

**Affiliations:** 1 Institute of Medical Chemistry, Biochemistry and Clinical Biochemistry, Faculty of Medicine, Sasinkova 2, Comenius University, 811 08 Bratislava, Slovakia; 2 Negentropic Systems, Murgasova 12, 03401 Ruzomberok, Slovakia

## Introduction

The simplest prediction related to our health is the one stating that if we don’t take care about our health and pay no attention to sustain it through the lifetime then we may expect that our health will get worse well before our late senior age.

In this communication we outline the way of how to estimate the health-related costs. Several attitudes are possible to answer the primary question. The WHO definition of health according to which ***“Health is a state of complete physical*, *mental and social well-being and not merely the absence of disease or infirmity” ***[1] indicates that we should make a complex assessment of costs of all factors influencing health.

Analyzing economic aspects of healthy life style is important from the point of view of predictive, preventive and personalized medicine which highlights preventive measures and personalized attitude towards the person who doesn’t want to become “a patient” but, on the other hand, he/she wants to know how much would it really cost to “be healthy” and whether it is affordable. The results of the analysis of all health-related costs have a potential for predicting health problems of the entire population on the basis of its economic status, as well as a potential for predicting health problems of an individual person.

## Components of health costs estimation

**Fig 1:**
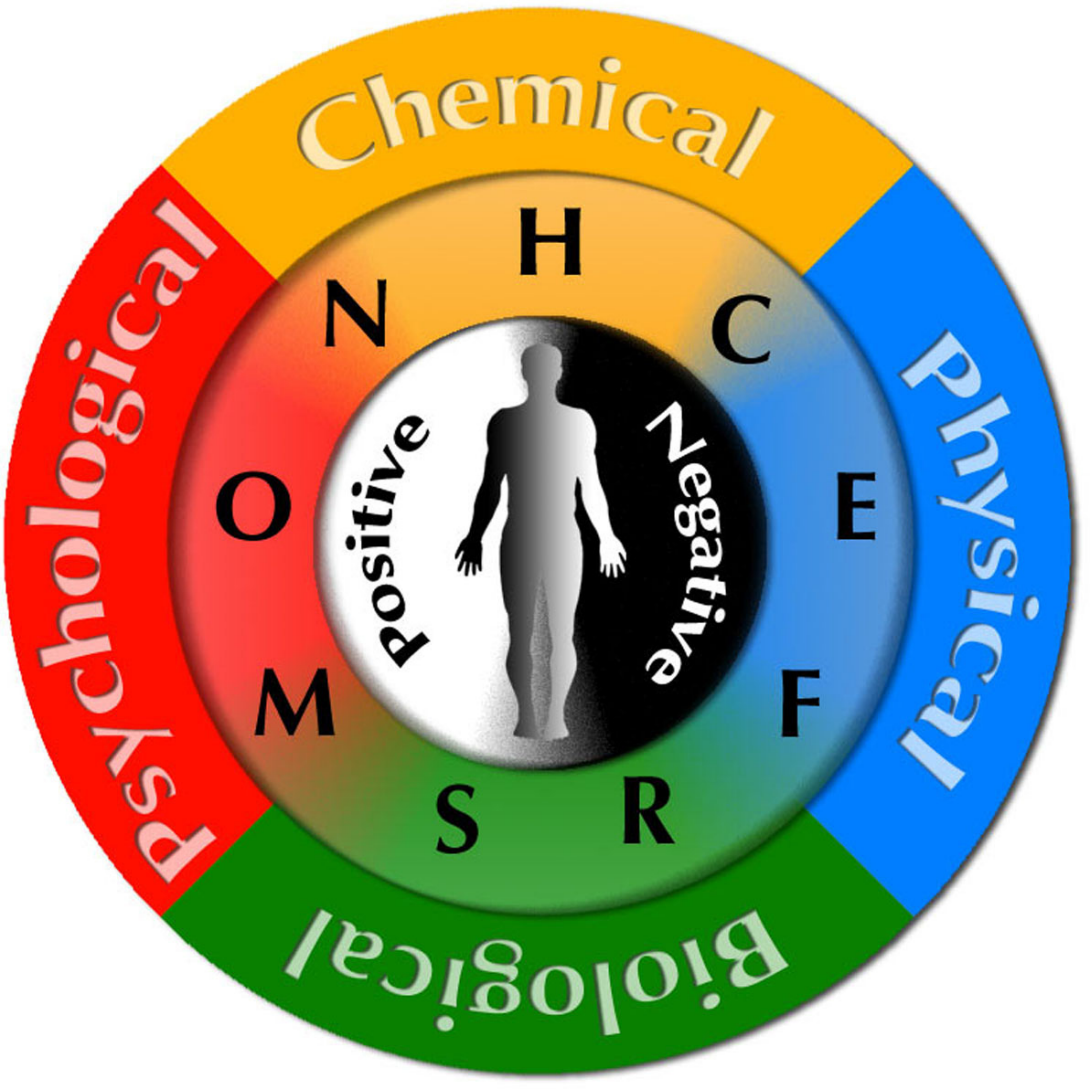
Factors influencing health, categorized according to prevailing nature of the factor. For the explanation of the particular letters see the text.

The health costs per person per time (e.g. month) should be calculated according to costs of the factors (determinants) which influence health (*general formula*). Basically, we can specify four categories of these factors, according to the prevailing nature of the factor (Fig. 1):

**• chemical,** all chemical substances influencing health in positive way (such as vitamins, minerals, water, air, etc) or negative way (toxic compounds and pollutants which body can not tolerate, such as those contained in low quality food, water, medical drugs, alcohol, tobacco products, cosmetics, furniture, clothing etc.)

**• physical,** all types of physical factors such a temperature, pressure, light, noise, sound, colors, gravity, magnetic field, electromagnetic field, radiation, vibration, and other

**• biological,** all types of biological entities ranging from viruses, bacteria, plants, animals to other humans, health of the person itself, genome, sleep, relax, including environment in general – forests, nature spots, national parks, and its proximity to housing

**• psychological,** all factors which are related to mental, emotional, cultural, social, financial, educational, spiritual and other having direct or facilitated influence on human mind or “*psyche*”

### General formula

Health costs per person per time = chemical + physical+ biological+ psychological

In each category we can determine factors which have **positive effect** on health as well as factors with negative influence. From the financial point of view the question is then how much are those positive factors and how much is the elimination of the **negative ones**.

For the practical purpose of calculating costs, however, we suggest *specific formula* which sums up concrete factors belonging to the one or more categories of the basic factors above. Such a formula also reflects the health determinants specified by World Health Organization [2], providing that we calculate the costs of sustaining or improving health of an otherwise “healthy” person. The particular costs may not necessarily reflect the real health impact and we also have to realize that the costs may be, to the certain extent, age and sex dependent.

### Specific formula

Health costs per person per month = N + H + C + E + F + R + S + M + O,

Where the letters stand for the following factors found to influence the health:

• Nutrition (N)

- costs of nutritionally balanced food (fruits, vegetables, meat, pasta, cereals, etc.) from both organic (certified products) and conventional food stores

• Hygiene and cosmetics (H)

- costs of all necessary products of standard hygiene and cosmetics, from both eco/bio/organic (certified products) stores and conventional stores

• Clothing (C)

- costs of all essential clothing made of organic fabrics and other technical fabrics

• Environment, nature, living (E)

- costs of living in an average city area, cost of “eco” living (healthy building, non-toxic furniture, electromagnetic shielding, noise elimination, darkness at night, fresh air, humidity), all running costs related to living such as heating, water, electricity, gas, sanitation, proximity of nature and transportation costs, cost of house/apartment ownership, waste disposal, etc.

• Family, society, education and philosophy (F)

- costs related to having family and friends, cost of education and communication (TV, internet, books, journals, phone, cell phone), costs related to a person’s philosophy (i.e. regular contributions for a foundation of choice, church, etc.)

• Relax, Sleep, Sport, Wellness, Culture (R)

- costs of different relax activities, including travel, massage, sauna, swimming pool, holiday, theatre, cinema, gallery, museum and other activities

• Social and economical stability (S)

- costs related to social insurance and other insurance, costs of having job, state support, taxes (car, house, highways, etc.), person’s savings

• Medicine, health-oriented healthcare, health insurance (M)

- these costs should reflect the costs related to minor health problems, entirely curable diseases, small injuries, health insurance, preventive measures, preventive procedures

• Other costs (O)

- All other costs that are not mentioned above but have impact on health.

We have to take into consideration also costs of sustaining or improving health of a person who suffers of any genetic disorder, chronic or rare disease. In many cases these cost may outweigh all other costs.

## Conclusion and recommendations

We recommend that particular estimation of all health-related costs according to the formula above is done for all countries. Consequently they can be compared to one another and used for particular evidence-based predictions and preventive actions, as well as for specific changes in the systems of healthcare.

We suggest the costs to be estimated for at least three levels, such as for example “*basic*” level, where only standard products and services are take into consideration, “*medium*” level, where there would be a combination of standard products and services and products of very high quality with positive effect on health (e.g., certified organic food, eco-certified cosmetics, bio-clothing, etc.) and “*high*” level, where all particular factors would be considered of high quality with appropriate certification.

To conclude, the calculation of health costs should not be solely about the “*healthy plate*” or “*organic farming*” as it may appear from the prevalent point of view presented in literature but rather it is a complex theme requiring multidisciplinary and integrative approach, the attitude which is shared also by predictive, preventive and personalized medicine. Undoubtedly, the complexity of this theme touches the very essence of the word “health” and has potential impact on the entire society and its philosophy for the lifetime.
